# The Use of Steroids to Treat Hypercalcemia Due to Granulomatous Disease From Disseminated Coccidioidomycosis

**DOI:** 10.7759/cureus.63977

**Published:** 2024-07-06

**Authors:** Tim Yu, Stephen Bruce, Diep Nguyen, Anika Patel, Mark MacElwee

**Affiliations:** 1 Department of Internal Medicine, Creighton University School of Medicine, Phoenix Regional Campus, Phoenix, USA; 2 Department of Internal Medicine, Creighton University School of Medicine, St. Joseph's Hospital and Medical Center, Phoenix, USA; 3 Department of Radiology, Creighton University School of Medicine, St. Joseph's Hospital and Medical Center, Phoenix, USA; 4 Department of Internal Medicine, Valleywise Health Medical Center, Phoenix, USA

**Keywords:** bisphosphonates, steroids, coccidioidomycosis, granulomatous disease, hypercalcemia

## Abstract

The clinical course and treatment of hypercalcemia from a granulomatous disease in the setting of an infectious etiology, namely disseminated coccidioidomycosis, remains incompletely understood. The mechanism and treatment of hypercalcemia have been documented in most granulomatous disorders, with sarcoidosis being the most well-understood so far. We discuss a case of a patient with a recent diagnosis of disseminated coccidioidomycosis who presented with hypercalcemia despite adequate infection control. The treatment course involved combinatorial-calcitonin, low-dose bisphosphonates, and corticosteroids, which led to a favorable outcome.

## Introduction

Coccidioidomycosis is a granulomatous fungal infection caused by the genus Coccidioides, which is endemic to the Southwestern United States; the majority of cases have been reported from Arizona (Phoenix and Tucson) and California (San Joaquin Valley). Over the past 30 years, the incidence of coccidioidomycosis in Arizona has increased considerably from 5.2 cases per 100,000 in 1990 to 160.6 cases per 100,000 in 2020 [1]. Coccidioides primarily grows in soil and infection occurs from the airborne inhalation of singular cells called arthroconidia [2]. While most patients remain asymptomatic after exposure, the clinical symptoms can range from pneumonia-like illness to extrapulmonary disseminated infection. The main risk factor for disseminated infection is cellular immunity suppression, and its extrapulmonary manifestations include meningitis, osteomyelitis, subcutaneous abscesses, and rarely, peritoneal involvement through lymphatic spread [3]. In rare cases, hypercalcemia may manifest in the setting of coccidioidomycosis. While hypercalcemia is a common finding in other granulomatous disorders like sarcoidosis [4] and tuberculosis [5], the mechanism of action and management for hypercalcemia in the setting of coccidioidomycosis is poorly understood. We present a patient with a rare case of hypercalcemia with low parathyroid hormone levels (PTH) and elevated parathyroid hormone-related peptide levels (PTHrP) in the setting of granulomatous coccidioidomycosis. We aim to discuss the clinical course and diagnostic challenges and highlight the use of steroids as a viable alternative to treat hypercalcemia in disseminated coccidioidomycosis.

## Case presentation

**Figure 1:**
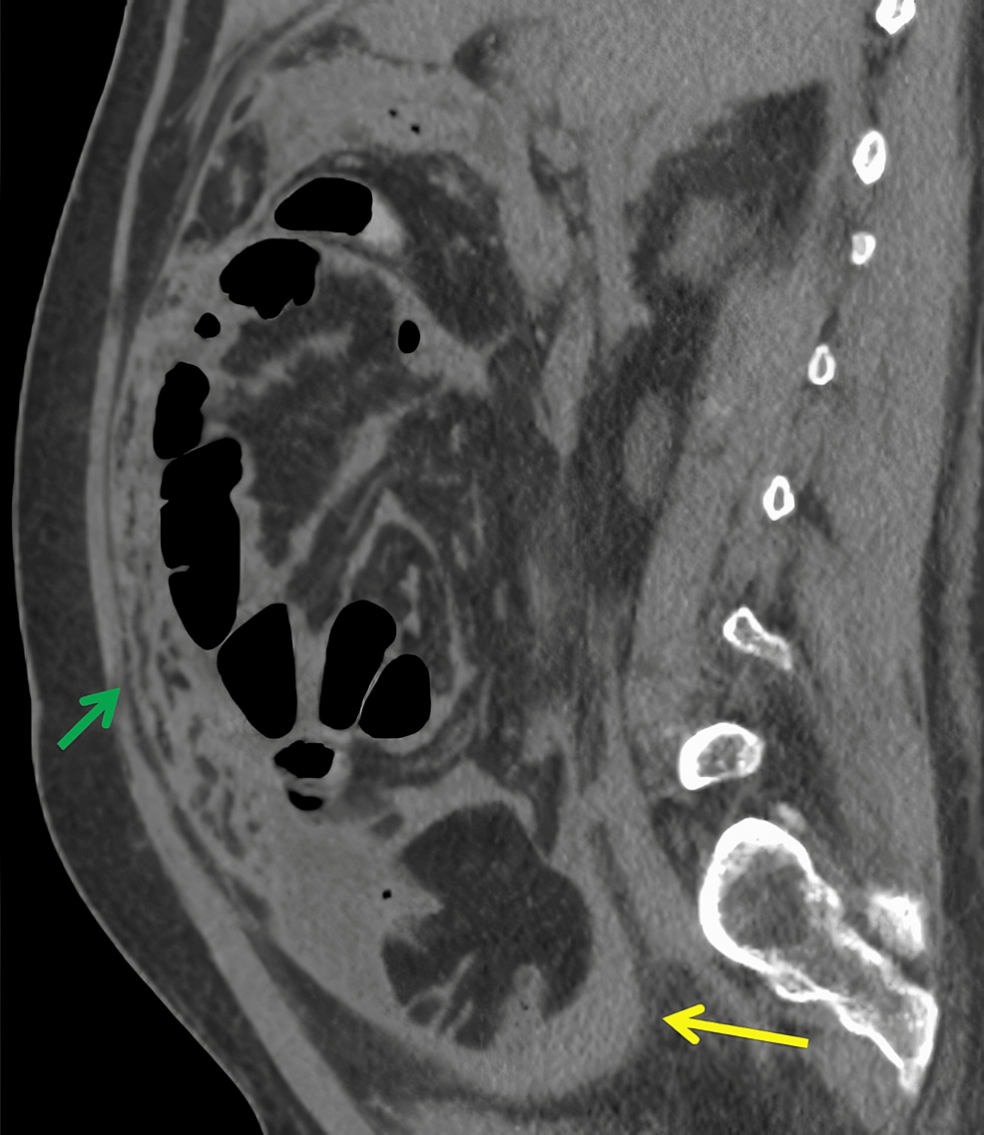
Sagittal non-contrast CT of the abdomen and pelvis The image shows scattered thickened mesenteric and omental nodularity (green arrow). In addition, there is a small amount of walled-off fluid in the pelvis (yellow arrow). The constellation of findings is concerning for peritonitis versus peritoneal carcinomatosis CT: computed tomography

**Figure 2:**
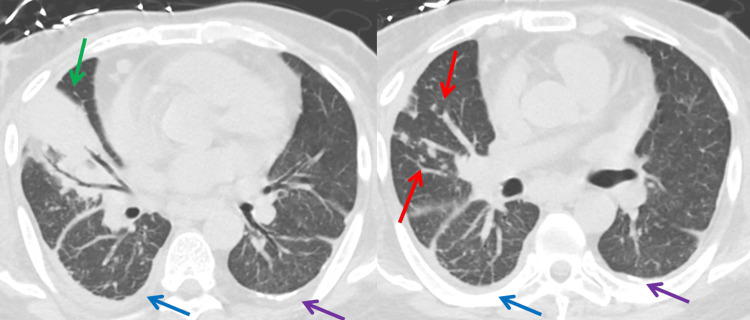
Axial non-contrast CT of the abdomen and pelvis The images show the middle (right panel) and lower (left panel) lung lobes. Right middle lobe consolidation (green arrows) with scattered centrilobular nodules throughout the right lung base(red arrows), trace right pleural effusion (blue arrows), and left pleural calcifications (purple arrows) can be seen CT: computed tomography

**Figure 3:**
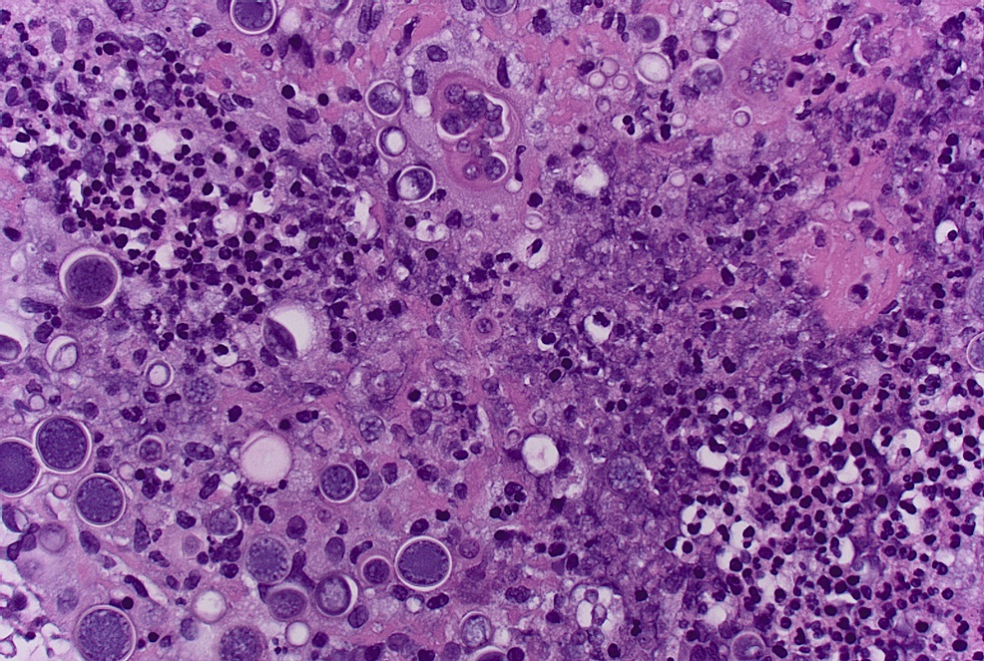
H & E stain of omental biopsy H & E stain showing thick-walled spherules with multiple endospores, suggesting coccidioidomycosis

**Figure 4:**
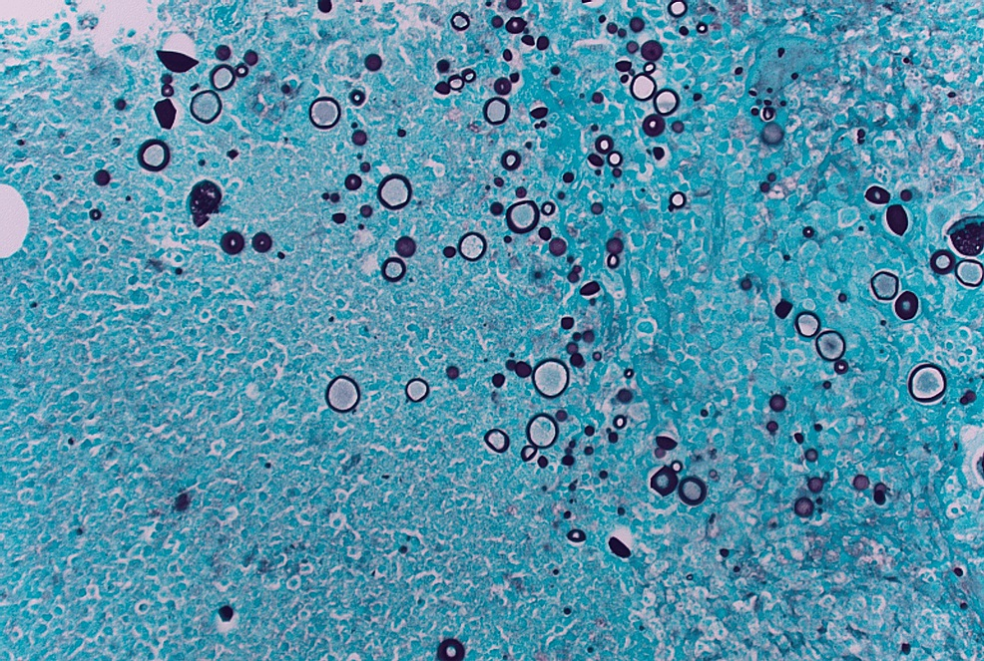
GMS stain of omental biopsy GMS-positive stain showing Coccidioides immitis spherules

A 41-year-old male with no significant past medical history presented to the hospital with three days of abdominal pain and a non-healing right calcaneal ulcer. MRI of the right ankle revealed acute osteomyelitis of the posterior calcaneus positive for methicillin-sensitive Staphylococcus aureus and Coccidioides. CT of the abdomen (Figures 1, 2) and subsequent omental biopsy (Figures 3, 4) at the time revealed granulomatous inflammation and coccidioidal peritonitis without malignancy, leading to a diagnosis of disseminated coccidioidomycosis.

Labs were obtained to further rule out malignancy and common causes of immunodeficiency. Serum IgG1 levels were elevated and IgA and complement levels were normal. CD4 count was 304 cells/μl (normal: 328-1404 cells/μl) and CD3 and CD8 counts were within normal limits. HIV serology was negative. Carcinoembryonic antigen, alpha fetal protein, and carbohydrate antigen 19-9 were all within normal limits. MRI of the brain showed no acute intracranial abnormality. The patient completed a six-week inpatient course of amphotericin B and was started on lifelong fluconazole therapy. While on amphotericin B, the patient developed acute tubular necrosis and was ultimately diagnosed with end-stage renal disease requiring hemodialysis. He showed improvement in his symptoms after the initiation of antifungals and was subsequently discharged after treatment completion. A repeat CT at the time of discharge showed improved pulmonary coccidioidomycosis with no new lesions.

One month after discharge, the patient returned to the hospital with abdominal pain and headaches after the dialysis center noted a calcium level of 15.9 mg/dL (normal: 8.4-10.2 mg/dL). The physical exam was positive for abdominal tenderness without distension or palpable masses. No focal neurological deficits were noted. At this admission, laboratory results revealed a white blood cell count of 7.2 K/uL (90.8% neutrophils, 3.4% lymphocytes, 3.9% monocytes, 0.5% eosinophils, and 0.2% basophils), hemoglobin of 9.5 g/dL, platelets of 263 K/uL, creatinine of 1.89 mg/dL (normal: 0.66-1.25 mg/dL), albumin of 2.7 g/dL (normal: 3.5-5 g/dL), phosphorus of 6 mg/dL (normal: 2.5-4.5 mg/dL), magnesium of 1.8 mg/dL (normal: 1.6-2.3 mg/dL), alkaline phosphatase of 62, PTH of 5.9 pg/mL (normal: 7.5-53.5 pg/mL), and low 25-OH-Vitamin D of 23.6 ng/mL (normal: 30-80 ng/mL).

The patient was started on 250 units of calcitonin twice per day and one dose of intravenous pamidronate 30 mg the following day with minimal improvement to his symptoms. Subsequent lab results showed 1,25-OH-Vitamin D of 175 pg/mL (normal: 19.9-79.3 pg/mL) and PTHrP of 3.9 pmol/L (normal: 0-2.3 pmol/L). Chest X-ray showed a small right pleural effusion with right middle lobe opacity. Tuberculosis had been ruled out based on negative acid-fast bacilli (AFB) cultures during the initial admission three months prior. Given the recent diagnosis of widely disseminated coccidioidomycosis, PTH suppression, elevated PTHrP, and markedly elevated active Vitamin D levels, he was diagnosed with hypercalcemia due to granulomatous disease from disseminated coccidioidomycosis. At this time, we decided to discontinue bisphosphonate therapy as he had not been responding to treatment, instead initiating a 10-day course of prednisone 60 mg/day, tapering 10 mg/day until 20 mg/day was reached. On this regimen, his symptoms resolved with the normalization of calcium levels. While malignancy is a reasonable differential due to elevated PTHrP, the lack of significant cancer biomarkers, histological samples, imaging findings, and the resolution of hypercalcemia following prednisone therapy make this less likely.

## Discussion

Disseminated coccidioidomycosis stands out as a rare and intriguing contributor to hypercalcemia, as its underlying mechanism and optimal treatment method remain elusive. Notably, our case aligns with the current literature that hypercalcemia associated with coccidioidomycosis is observed only in the disseminated form. A study involving 13 patients by Caldwell et al. found no correlation between elevated 1,25-dihydroxyvitamin D and hypercalcemia due to coccidioidomycosis, which contrasts with our case and known mechanisms such as those in sarcoidosis [6]. Another study by Fierer et al. suggested that granuloma PTHrP overexpression may contribute to hypercalcemia in disseminated coccidioidomycosis, consistent with our patient's case [7]. Nonetheless, larger and more extensive studies are needed to fully elucidate the mechanism behind hypercalcemia in disseminated coccidioidomycosis.

The management of hypercalcemia in granulomatous diseases, notably studied in sarcoidosis, usually involves moderate-dose steroid therapy [8]. In refractory conditions or steroid intolerance, bisphosphonates, chloroquine, and anti-TNF alpha therapy have shown efficacy [8-10]. Granulomatous hypercalcemia caused by infections typically resolves with successful infection treatment. Data are scarce on the treatment of patients who suffer from this condition despite adequate infection control, such as our patient, who had already undergone a six-week course of amphotericin B and was on lifelong fluconazole when hypercalcemia manifested. Bisphosphonate therapy has been shown to successfully resolve granulomatous hypercalcemia due to disseminated coccidioidomycosis [7,11]. The most commonly prescribed bisphosphonate therapy is pamidronate 60 mg. To our knowledge, there is only one case report documenting the favorable use of steroids, which involved administering one dose of hydrocortisone 10 mg followed by 5 mg [12].

Traditionally, glucocorticoids have been avoided in treating granulomatous diseases from infectious origins due to concerns about immune suppression [13]. Further studies are necessary to assess the potential risk of corticosteroids inducing exacerbations of disseminated coccidioidomycosis on adequate prophylaxis. In our case, glucocorticoids proved effective in resolving hypercalcemia associated with disseminated coccidioidomycosis without subsequent acute exacerbations of the disseminated infection. While a higher and more prolonged dose of bisphosphonate therapy might have proven effective, glucocorticoids should be considered as an effective alternative in treating patients with hypercalcemia due to granulomatous disseminated coccidioidomycosis.

## Conclusions

Disseminated coccidioidomycosis is a relatively rare cause of hypercalcemia, and more serious causes such as malignancy must be ruled out in these patients. A diagnosis was made in our patient based on a combination of histological, radiological, clinical, and laboratory findings. Given the limited evidence on treating granulomatous hypercalcemia in disseminated coccidioidomycosis, we aim to underscore the effective use of steroids in this context, offering providers an alternative treatment option.
